# Exploring Sociodemographic Characteristics, Adverse Childhood Experience, and Mental Health History as Predictors of Anxiety and Depression among Adolescents and Young Adults: Findings from the MoreGoodDays Support Program in Alberta, Canada

**DOI:** 10.3390/bs13090749

**Published:** 2023-09-08

**Authors:** Belinda Agyapong, Reham Shalaby, Katherine Hay, Rachal Pattison, Ejemai Eboreime, Mark Korthuis, Yifeng Wei, Vincent Israel Opoku Agyapong

**Affiliations:** 1 Department of Psychiatry, Faculty of Medicine and Dentistry, University of Alberta, Edmonton, AB T6G 2B7, Canada; 2 Kickstand, Edmonton, AB T5K 2J8, Canada; 3 Department of Psychiatry, Faculty of Medicine, Dalhousie University, Halifax, NS B3H 4R2, Canada; 4 Glenrose Rehabilitation Hospital Foundation, Edmonton, AB T5G 0B7, Canada

**Keywords:** anxiety, depression, adolescents, young adults, mental health counselling, MoreGoodDays

## Abstract

Background: The COVID-19 pandemic has increased psychological disorders among adolescents and young adults. Methods: This study used a cross-sectional design. An online survey questionnaire was used to collect sociodemographic and clinical information from subscribers of MoreGoodDays program, a daily supportive text message program co-designed with adolescents and young adults for their peers in Alberta. Validated instruments, the Generalized Anxiety Disorder GAD-7 scale and Patient Health Questionnaire-9 PHQ-9 were used to collect information on likely GAD and likely major depressive disorder (MDD). Data was analyzed with SPSS version 25 using chi-squared tests and binary logistic regression analysis. Results: 343 subscribers of MoreGoodDays participated in the survey. Overall, 117 (56.0%) respondents had a likely MDD and 97 (46.6%) had a likely GAD. Participants who would like to receive mental health counselling were 27 times more likely to experience GAD (OR = 27; 95% CI: 3.09–250.00) and 40 times more likely to experience MDD (OR = 40.03; 95% CI: 4.43–361.51) than those who did not. Respondents who had received mental health counselling in the past were 18.5 times more likely to experience MDD compared with those who had not (OR = 18.52; 95% CI: 1.55–200.00). Demographic variables, including age, education, employment, and relationship status, and clinical variables, such as history of anxiety, depression, obsessive-compulsive disorder, ADHD, and adverse childhood experience, did not independently the predict presence of likely GAD or MDD in subscribers of MoreGoodDays. Conclusion: The prevalence of anxiety and depression was relatively high among subscribers of MoreGoodDays, indicating the long-term effect of the COVID-19 pandemic. This finding has significant implications in the broader contextof mental health research and emphasizes the need for more research into innovative mental health support for this cohort. The desire to receive counselling was predictive of both anxiety and depression and is a positive sign of the openness of this cohort to receive psychological intervention. Since this group is mostly adapted to mobile text technology, government agencies and policymakers should prioritize and implement readily accessible interventions such as supportive text messages to support their psychological well-being.

## 1. Introduction

Adolescents and young adults exhibit an increasing prevalence of mental health problems, specifically depression and anxiety, which often coexist [1,2,3]. The occurrence of one condition typically heightens the risk of the other over time [4]. Anxiety, characterized by a diffuse emotional state anticipating potential harm or uncertain threats, poses a significant health issue for this age group [5,6,7]. Notably, a study conducted during the COVID-19 pandemic reported a 75% incidence of moderate anxiety among young adults aged 21 to 25 years, a critical transition stage marked by physiological and psychological changes that may result in loneliness leading to depression as well as low self-esteem [8,9]. Additionally, a global study across 82 countries found anxiety prevalence among adolescents ranging from 7% to 12%, with the highest pooled prevalence of 17.0% [10]. However, among the general public, a systematic review and meta-analysis of over 2 million people during the pandemic reported an anxiety prevalence of 35.1% [11].

Various factors contribute to increased anxiety levels among adolescents and young adults, including academic stressors, unemployment, financial and societal stress, course workload, and socioeconomic status [10,12,13]. Insufficient sleep hours have also been associated with higher anxiety among adolescent students [13]. Depression, on the other hand, typically increases from late childhood through early adulthood [14]. During the COVID-19 pandemic, one-fifth of young adults and a third of adolescents had mental health issues, including depression and generalized anxiety disorder [15]. Depression is particularly prevalent among young adults, with a notable surge during adolescence, and is linked to adverse mental health outcomes, economic factors, anxiety disorders, suicidal ideation, and unemployment [16,17,18,19,20].

Gender differences have been reported in the literature with females inadvertently impacted by mental health issues more than males. Females, for instance, exhibited a higher prevalence of major depressive disorder [20].

The COVID-19 pandemic has further amplified mental health vulnerabilities among young adults and adolescents, especially females [14,21]. However, studies suggest that symptoms of anxiety and depression were already elevated even before the pandemic, with students experiencing higher levels of stress, anxiety, and depression on average [22]. A multi-country survey indicates that socioeconomic status and geographical region influence anxiety levels among young adults and adolescents [23], complicating the magnitude of mental health issues. Furthermore, anxiety and depression have detrimental effects on educational achievement, emotional and social functioning, self-esteem, quality of life, and general well-being [24,25]. Comorbidity between depression and anxiety is common, emphasizing the need for targeted interventions to reduce the likelihood of experiencing either condition [4,26]. Accessible, low-cost public health interventions are crucial for addressing these challenges [26]. Nevertheless, a gap exists in the type and mode of delivery of mental health interventions that are technologically inclined and may be appealing to young adults and adolescents.

E-communication technologies, such as instant messaging are highly prevalent among adolescents and young adults [27]. Leveraging this trend, the MoreGoodDays program was developed as an evidence-based text and email messaging innovation to support young individuals’ mental health of young individuals [28,29]. Crafted by a collaboration between Kickstand, Alberta, the Global Mental Health Research Group, the University of Alberta, and Dalhousie University, the program offers free daily text messages in Alberta when individuals subscribe by texting ‘MoreGoodDays’ to 393939 [28]. Operating similarly to the Text4Hope service implemented during the COVID-19 pandemic in Alberta, MoreGoodDays delivers cognitive–behavioral therapy-based supportive messages to subscribers’ cell phones for twelve months [26,30,31]. Subscribers receive different messages daily that may identify with their struggles or concerns or offer advice or solutions. It is important to note that the messages delivered through MoreGoodDays were written by young adults, reviewed by a clinical team, and revised as needed to alleviate anxiety and depression symptoms among adolescents and young adults. This distinction sets MoreGoodDays apart from other ResilienceNHope programs, such as Wellness4Techers and Text4Hope [32,33]. MoreGoodDays program will potentially help address the identified mental health issues such as anxiety and depression and benefit the adolescents and young adults who are technologically inclined and hence are more likely to assess this text messaging program. The aim of this study is to assess the prevalence, severity, and correlates of likely generalized anxiety disorder (GAD) and likely major depressive disorder (MDD) among subscribers of the MoreGoodDays program. Considering the enormous global impact of the pandemic, these prevalence estimates will be significant in appreciating the impact of the pandemic among adolescents and young adults, specifically in the Alberta context.

## 2. Methodology

### 2.1. Study Settings and Design

This study was conducted in Alberta, Canada. Alberta is one of the fastest growing of the 13 provinces and territories in Canada, with a population of 4,262,645 as per the 2021 census [34]. In order to assess the prevalence estimates of anxiety and depression in subscribers of the MoreGoodDays, a cross-sectional design was adopted. A cross-sectional design was chosen as this suited the research objectives, and the focus was on a representative subset of the population: adolescents and young adults.

### 2.2. Ethics Statement and Consent

Study approval was granted by the Alberta Health Research Ethics Committee (Pro00106957). The online survey began with information about the study and included statements advising participants that consent was implied if they completed and submitted the online survey. Respondents’ privacy and data protection were assured throughout the study as no participant-identifiable data was collected.

### 2.3. Data Collection and Outcome Measurement

Subscribers of MoreGoodDays (participants) were invited via text messages to complete voluntary online survey questionnaires at baseline (on subscription). Data was collected between 28 January 2021 to 17 July 2022. The online survey link was sent to subscribers at baseline, six weeks, three months, and six months. The survey included a blend of categorical sociodemographic and self-reported clinical questions, including age, gender, ethnicity, employment status, educational level, marital status, housing status, adverse childhood experience, history of mental health conditions, treatment received, counselling received or willingness to receive, history of anxiety disorder, and history of depressive disorder. We assessed likely generalized anxiety disorder (GAD) and likely major depressive disorder (MDD) among MoreGoodDays subscribers using the Generalized Anxiety Disorder-7 (GAD-7) scale [35,36] and the Patient Health Questionnaire-9 (PHQ-9) [37], respectively. A GAD-7 score of ≥10 indicates likely GAD [35], whilst a PHQ-9 score of ≥10 indicates likely MDD [37].

As of 17 July 2022, there were 1045 active subscribers of the MoreGoodDays service, with a 95% confidence interval and a ±3% margin of error. The sample size needed for prevalence estimates for anxiety symptoms would be 529. The margin of error and confidence interval were chosen based on those used in sample size estimation in publications [38].

### 2.4. Statistical Analysis

Data were analyzed using SPSS Version 25 (IBM Corp 2011, Armonk, NY, USA) [39]. For the prevalence study, descriptive statistics were summarized for demographic and clinical variables, including prevalence estimates based on the age of the respondents. We examined all the variables for association with likely GAD and MDD and conducted chi-squared analysis. Two independent binary logistic regression analyses were used to identify variables that were independently predictive of likely GAD and MDD. The regression model included variables which were significantly (*p* < 0.05) or nearly significantly (0.1 ≥ *p* ≥ 0.05) associated with likely GAD and likely MDD in chi-square analysis. Prior to the regression analysis, correlational analysis was executed to exclude any strong intercorrelations (Spearman’s correlation coefficient of 0.7 to 1.0 or −0.7 to −1.0) among the predictors. Odds ratios (OR) and confidence intervals were reported, determining the predictor variables to self-report likely GAD and likely MDD. We also controlled for other variables in the two models. Reported results represent measured prevalence and severity of MDD and GAD, using frequency/percentages and mean scores, respectively, while using odd ratios (OR) and confidence intervals to report the correlates of the clinical conditions. There was no imputation for missing data.

## 3. Results

### 3.1. Descriptive Analysis

Table 1 shows the sociodemographic characteristics of the participants. Overall, 343 out of 1045 subscribers of MoreGoodDays participated in the baseline survey, representing a response rate of 32.8%. Majority of the respondents were females (271, 79.0%). Most of the participants were white (250, 73.1%), employed (163, 47.4%), and lived with family and friends (148, 43.0%), with about 106 (30.8%) owning a home. About 170 (49.4%) were single, and 155 (45.1%) were in a relationship (married, common law, partnered). More than half of the respondents, i.e., 214 (62.2%), reported they were on no psychotropic medications. A mental health history of depression and anxiety was observed in 124 (36.0%) and 135 (39.2%) respondents, respectively, and about (64, 35.4%) of respondents expressed the desire to receive mental health counselling. Only 54 respondents (24.8%) reported no adverse childhood experiences and about a third (72, 33%) reported four or more adverse childhood experiences. Overall, 117 (56.0%) of respondents met the criteria for likely MDD and 97 (46.6%) met the criteria for likely GAD.

### 3.2. Predictors of Likely GAD

#### 3.2.1. Univariate Analysis

Table 2 shows the results of chi-square test of association between demographic, clinical, and mental health characteristics and likely GAD disorder. The results in Table 2 indicate that, overall, 15 variables had a statistically significant association with likely GAD, including age, educational levels, relationship status, employment status, housing status, ACE score, mental health history of depression, anxiety, OCD, and ADHD, no mental health history, on antidepressants, on sleeping tablets, on psychotropic medication, and desire to receive mental health counselling. Participants aged 26 years or less, in high school, were students, were employed, were separated/divorced/widowed, and were living in rented accommodation had higher prevalence of likely GAD compared to other participants in their respective categories. Similarly, participants with an increasing number of adverse childhood traumas and history of depression, anxiety disorder, OCD, and ADHD, those with no mental health history, those on an antidepressant, sleeping tablets, or psychotropic medication, and those who either sought or would like to receive mental health support had a higher prevalence of likely GAD compared with other participants in their respective categories.

#### 3.2.2. Logistic Regression Analysis to Identify Predictors of Likely GAD

Overall, 12 predictors that had a significant association or a trend towards significant association with likely GAD in chi-square/Fisher’s exact test were included in a binary logistic regression model. Three other variables were highly correlated (r ≥ 0.7) with other variables on correlation analysis and were excluded from the regression analysis. The excluded variables included “housing status”, which was highly correlated with “age”, “have never received mental health diagnosis”, which was highly correlated with “diagnosis of anxiety disorder”, and “not on any psychotropic medication”, which was highly correlated with “on an antidepressant”. In each case, the rationale for excluding the former rather than the latter from the logistic regression model was that the latter variables were thought to be more relevant to the outcome of interest.

Table 3 shows the results for the logistic regression model for likely GAD. The model was statistically significant; *Χ*^2^ (df = 20; *n* = 351) = 49.20, *p* < 0.000, suggesting that the logistic regression model could differentiate between MoreGoodDays participants with likely GAD or not. The model explained 35.8% (Cox and Snell R^2^) to 48.1% (Nagelkerke R^2^) of the variance and correctly classified 79.3% of cases. Table 3 shows that only one variable, “Would you like to receive mental health counselling?” independently predicted the likely presence of GAD in the MoreGoodDays participants. After controlling for all other variables in the regression model, participants who would like to receive mental health counselling were 27 times more likely to experience GAD symptoms than those who did not (OR = 27; 95% CI: 3.09–250.00). Other demographic and clinical variables such as age, history of adverse childhood experience, and history of anxiety or depressive disorder did not independently predict the presence of likely GAD in subscribers of the MoreGoodDsys program.

### 3.3. Predictors of Likely MDD

#### 3.3.1. Univariate Analysis

Table 4 shows the results of the chi-square test of the association between demographic, clinical, and mental health characteristics and likely MDD. Table 4 below indicates that, overall, 16 variables had a statistically significant association in chi-square/Fisher’s exact test (*p* < 0.05) or near significant (0.1 ≥ *p* ≥ 0.05) with likely MDD, including age, educational level, relationship status, employment status, housing status, ACE score, mental health history of depression, anxiety, SUD, ADHD, no mental health history, on antidepressants, on sleeping tablets, on no psychotropic medication, and those who have received mental health counselling or desire to receive mental health counselling. Participants who were aged 26 years or less, had an education level of less than high school, were single, were students and employed, were living in rented accommodation or with family or friends and had an ACE score of three had a higher prevalence of likely MDD relative to other participants in their respective categories. Similarly, participants with a history of depression, GAD, SUD, ADHD, those with no mental health history, those on antidepressants, those on no sleeping tablets, and MoreGoodDays participants who had received mental health counselling or would like to receive mental health counselling or were unsure if they would like to receive mental health counselling had a higher prevalence of likely MDD relative to other MoreGoodDays participants in their respective categories.

#### 3.3.2. Logistic Regression Analysis to Identify Predictors of Likely MDD

Overall, 13 predictors that had a significant association or a trend towards significant association with likely MDD in chi-square analysis were included in a binary logistic regression model. Three other variables that were highly correlated with some of the included variables of correlation analysis were excluded from the regression model. The excluded variables included “housing status”, which was highly correlated with “age”, “receipt of mental health diagnosis”, which was highly correlated with “diagnosis of anxiety disorder”, and “not on any psychotropic medication”, which was highly correlated with “on an antidepressant”. In each case, the rationale for excluding the former variables rather than the latter ones from the logistic regression model was that the latter variables were thought to be more relevant to the outcome of interest. The logistic regression model was statistically significant; *Χ*^2^ (df = 21; *n* = 351) = 67.75, *p* < 0.000, suggesting that the model could differentiate between MoreGoodDays subscribers with likely MDD and those without. The model explained 45.7% (Cox and Snell R^2^) to 60.9% (Nagelkerke R^2^) of the variance and correctly classified 82.9% of cases. Table 5 shows that two variables, “Have received mental health counselling in the past” and “would like to receive mental health counselling?” independently predicted the likely presence of MDD in MoreGoodDays subscribers. The results suggest that MoreGoodDays participants who had received mental health counselling in the past were 18.5 times more likely to experience MDD symptoms compared with those who had not (OR = 18.5; 95% CI: 1.55–200.00). Similarly, MoreGoodDays participants who would like to receive mental health counselling were 40 times more likely to experience MDD than those who did not (OR = 40.03; 95% CI: 4.43–361.51). Again, MoreGoodDays participants who were undecided or unsure if they would like to receive mental health counselling were 29 times more likely to experience MDD than those who did not (OR = 28.86; 95% CI: 3.07–271.45). Other important predictor variables, such as age, history of adverse childhood experience, and history of anxiety or depressive disorder, did not independently predict the presence of likely MDD in this model.

## 4. Discussion

Young adults have been disproportionately affected by mental health issues, particularly anxiety and depression, during the pandemic [40,41]. Our study aimed to assess the prevalence and predictors of likely generalized anxiety disorder (GAD) and major depressive disorder (MDD) among subscribers of MoreGoodDays, a daily supportive text message program co-created with adolescents and young adults for their peers. The objective was to be conversant with this estimated prevalence among adolescents and young adults, specifically in Alberta.

### 4.1. Prevalence of GAD and MDD

In our study, the prevalence of likely GAD among respondents aged 26 years or younger was 55.1%, compared to 37.1% for those older than 26 years. The overall prevalence of likely GAD in the study sample was 46.6%, which is relatively higher than prevalence estimates reported in studies conducted before the COVID-19 pandemic, with the highest pooled prevalence of 17.0% among adolescents [10]. The prevalence of anxiety in our study is comparable to prevalence estimates reported in other studies conducted during the pandemic. For example, a cross-sectional study in North America reported that 48.1% of students had symptoms of anxiety [42]. Similarly, other studies during the pandemic reported a higher prevalence of anxiety, such as 60.8% among health science students [43] and 66.86% among other respondents [44].

The overall prevalence of likely MDD in our respondents was 56%, with a prevalence of 69.6% among participants aged 26 years or younger. These rates are higher than what was reported in other studies regarding impacts on mental health issues. For instance, a study among young adults before the COVID-19 pandemic recorded a prevalence of (65%) for depression [45]. Our findings add to the existing literature, suggesting that anxiety and depression are prevalent among adolescents and young adults and confirm the impact of the pandemic on this cohort [22]. In addition, another study reported a prevalence of 57.39% among student participants in a cross-sectional study conducted in Asia during the pandemic [43], which corroborates our study with an overall likely MDD prevalence of 56%. Furthermore, our study supports previous research showing no significant decrease in the 12-month prevalence of depression from ages 21 to 30 [46] and a higher prevalence of anxiety and depression among the adolescent and youth group during the pandemic [14]. The potential reasons behind higher prevalence rates of anxiety and depression in our study compared to pre-pandemic estimates are that the pandemic increased uncertainty worldwide and caused short-term confinement, isolation, and travel restrictions, at least in the initial stages. The imposed behavioural changes during the COVID-19 pandemic may have also contributed to the increased number of adolescents and young adults with likely anxiety and likely depressive symptoms and other disorders at levels that require treatment. Other studies during the pandemic also reported relatively higher prevalence rates compared to the pre-pandemic era. Other possible grounds for variations include differences in demographics, such as age and gender, methods used for data collection, and assessment tools. Females have been reported to be disproportionally impacted by mental health issues more than males [20,47]. However, our study reported a slightly higher prevalence of anxiety (51.5%) and likely MDD (57.6%) in males. This is consistent with a study that reported a high prevalence of 53.8% among male students before the pandemic [48].

### 4.2. Predictors of GAD and MDD

In our study, demographic variables, such as age, education, employment, and relationship status, as well as clinical variables, including anxiety, depression, obsessive-compulsive disorder, attention deficit hyperactivity disorder (ADHD), and adverse childhood experiences (ACE), did not independently predict likely GAD or MDD. This finding aligns with another study that did not find independent predictors of suicidal ideation and self-harm among participants [49]. However, it contradicts a different study suggesting a statistically significant association between anxiety level and participant age [8]. Age was also found to be a significant predictor of moderate-to-high anxiety among the general public in a study conducted in Canada during the pandemic, with the cohort aged 25 years or younger more likely to experience moderate-to-high anxiety [50]. Relationship status, which is considered an important factor in mental health and well-being, did not predict anxiety or depression in our study. Similarly, employment status did not independently predict anxiety or depression. However, a longitudinal study reported a significant association between depression and unemployment [18]. This longitudinal study also found that depression diagnosis independently predicted the presence of likely GAD and that the frequency of depressive episodes experienced by adolescents and young adults predicted later psychiatric outcomes, including anxiety and depression [18]. In contrast to these reports, our study found that a history of mental health conditions, including anxiety and depression, did not independently predict the presence of likely GAD or MDD in subscribers of MoreGoodDays.

Adverse childhood experiences have long been linked to various mental health issues and may have a lifelong effect on an individual’s mental and physical health [51]. For example, one study indicated a strong link between ACE and an ADHD diagnosis in later life [52], as well as higher risks of depression that persist after adjustment for family and socioeconomic factors [53]. Another study reported that experiencing four or more categories of adverse childhood experiences leads to a four to twelve-fold increase in health issues such as depression [54]. Despite the association reported in the literature between ACE and mental health issues in adolescents and young adults, this study failed to demonstrate this association with depression or anxiety.

This study revealed that the desire to receive mental health counseling is the only variable out of the twelve variables examined that independently predicts likely GAD among subscribers of MoreGoodDays. Participants who expressed a desire for mental health counseling were 27 times more likely to experience GAD symptoms than those who did not. Similarly, participants who desired mental health counseling were 40 times more likely to experience MDD symptoms. In contrast, those who were undecided or unsure about counseling were 28.9 times more likely to experience MDD symptoms. Probable underlying reasons for this may be the increased awareness of mental health issues during the pandemic. Desire to receive mental health counseling has been associated with an increased likelihood of other mental health issues. For example, a study reported that individuals who desired to receive mental health counseling were seven times more likely to report suicidal ideation and thoughts of self-harm relative to participants who did not desire counseling [49]. A cross-sectional study among college students during the COVID-19 pandemic also found that individuals with anxiety and depression symptoms significantly desired more psychological knowledge and interventions [55]. The desire to receive mental health counseling may indicate psychological distress and the need for intervention. Other studies proposed that the desire for mental health counseling may offer better insight into possible mental distress than individual clinical variables, necessitating the importance of providing prompt online psychological counseling services during the pandemic [49,56].

Finally, this study found that participants who had received mental health counseling in the past year were 18.5 times more likely to experience MDD symptoms than those who had not. Individuals who have received counselling in the past year may have underlying psychological problems or vulnerability factors that make them prone to develop mental disorders. A systematic review [57] reported that therapy duration varies from one hour to longer than 20 h; hence, the duration of counseling received by individuals, especially if sessions are not complete, may increase vulnerability. Additionally, there seems to have been an increased awareness of mental health issues during the pandemic, which was a positive outcome, but this may have led to the increased desire to access counselling. However, it is essential to note that the type and duration of counseling received by participants in the past year were not specified or explored in our study, making it difficult to interpret this finding. Cognitive–behavioral therapy (CBT) has shown promise in mitigating mental health illnesses such as anxiety and depression [58] among the general public though we could not stipulate if CBT was specifically adopted by our study participants.

### 4.3. Study Limitations

This study has some limitations worth mentioning. First, the scales used to assess mental health variables, although standardized, are not diagnostic, making it impossible to determine the proportion of the sample experiencing transient symptoms versus clinically significant disorders. Second, data in this study were collected through a self-report online questionnaire, and while standardized assessment scales were used, they were not diagnostic. Third, the demographic variables in the study did not reflect the demographics of Alberta’s adolescent and youth population.. With the majority of our study sample being females (more than 70%) and less than 14% being male participants, the findings may not be generalizable to the intended target population of the MoreGoodDays program.

Additionally, although the MoreGoodDays program was co-designed with adolescents and young adults, a significant number of subscribers were adults. Finally, data was collected from subscribers of the MoreGoodDays program who may consider themselves to need mental health care and, therefore, may have biased the study results. Despite these limitations, our study provides valuable information about the prevalence of anxiety and depression during the COVID-19 pandemic and highlights the long-term impact on adolescents and young adults. These findings have implications for government agencies and policymakers working with these populations.

## 5. Conclusions

The COVID-19 pandemic has increased the vulnerability of adolescents and young adults to psychological disorders such as anxiety and depression. Our study demonstrates that the desire for mental health counseling is the only variable that independently predicts likely GAD among subscribers of the MoreGoodDays program. Participants who expressed a desire for counseling were significantly more likely to experience GAD and MDD symptoms. The high prevalence of anxiety and depression among our study participants confirms the long-lasting effects of the pandemic on this population. Our study results significantly contribute to the existing knowledge on the mental health impact of the pandemic. Text messaging interventions like MoreGoodDays may help reduce the treatment gaps in mental health support for adolescents and young adults, especially considering their affinity for mobile text technology. MoreGoodDays intervention is based on mobile text messaging.

Furthermore, other comprehensive mental health literacy programs could be incorporated as part of the educational curriculum by policymakers to support the mental health needs of adolescents and young adults. Government agencies and policymakers should prioritize and implement accessible interventions, such as supportive text messages, to support the psychological well-being of vulnerable populations [26,59]. There should also be ongoing advocacy to highlight the importance of mental health support for young adults, even after the pandemic.

## Figures and Tables

**Table 1:** Sociodemographic, clinical, and mental health related variables distributed based on age.

Variables	≤26 Years n (%) N =	>26 Years n (%) N =	Total n (%) N =
**Sociodemographic characteristics**			
**Gender**			
Male	24 (13.2%)	23 (14.3%)	47 (13.7%)
Female	136 (74.7%)	135 (83.9%)	271 (79.0%)
Other	22 (12.1%)	3 1.9%)	25 (7.3%)
**Ethnicity**			
White	114 (63.0%)	136 (84.5%)	250 (73.1%)
Aboriginal	23 (12.7%)	14 (8.7%)	37 (10.8%)
Asian	28 (15.5%)	7 (4.3%)	35 (10.2%)
Other	16 (8.8%)	4 (2.5%)	20 (5.8%)
**Educational level**			
Less than high school	43 (23.5%)	7 (4.3%)	50 (14.5%)
High school	65 (35.5%)	10 (6.2%)	75 (21.8%)
Postsecondary education	75 (41.0%)	144 (89.4%)	219 (63.7%)
**Relationship status**			
In a relationship (married, common law, partnered)	43 (23.5%)	112 (69.6%)	155 (45.1%)
Single	139 (76.0%)	31 (19.3%)	170 (49.4%)
Separated/divorced/widowed	1 (0.5%)	18 (11.2%)	19 (5.5%)
**Employment status**			
Employed	41 (22.4%)	122 (75.8%)	163 (47.4%)
Unemployed	16 (8.7%)	23 (14.3%)	39 (11.3%)
Student	77 (42.1%)	5 (3.1%)	82 (23.8%)
Student and employed	49 (26.8%)	11 (6.8%)	60 (17.4%)
**Housing status**			
Own home	5 (2.7%)	101 (62.7%)	106 (30.8%)
Rented accommodation	39 (21.3%)	51 (31.7%)	90 (26.2%)
Live with family or friend	139 (76.0%)	9 (5.6%)	148 (43.0%)
**MH history**			
**Depression**			
No	103 (56.3%)	117 (72.7%)	220 (64.0%)
Yes	80 (43.7%)	44 (27.3%)	124 (36.0%)
**BD**			
No	179 (97.8%)	155 (96.3%)	334 (97.1%)
Yes	4 (2.2%)	6 (3.7%)	10 (2.9%)
**GAD**			
No	90 (49.2%)	119 (73.9%)	209 (60.8%)
Yes	93 (50.8%)	42 (26.1%)	135 (39.2%)
**Eating disorder**			
No	167 (91.3%)	150 (93.2%)	317 (92.2%)
Yes	16 (8.7%)	11 (6.8%)	27 (7.8%)
**OCD**			
No	166 (90.7%)	149 (92.5%)	315 (91.6%)
Yes	17 (9.3%)	12 (7.5%)	29 (8.4%)
**SUD**			
No	181 (98.9%)	155 (96.3%)	336 (97.7%)
Yes	2 (1.1%)	6 (3.7%)	8 (2.3%)
**Schizophrenia**			
No	182 (99.5%)	160 (99.4%)	342 (99.4%)
Yes	1 (0.5%)	1 (0.6%)	2 (0.6%)
**PD**			
No	179 (97.8%)	154 (95.7%)	333 (96.8%)
Yes	4 (2.2%)	7 (4.3%)	11 (3.2%)
**ADHD**			
No	172 (94.0%)	152 (94.4%)	324 (94.2%)
Yes	11 (6.0%)	9 (5.6%)	20 (5.8%)
**PTSD**			
No	156 (85.2%)	138 (85.7%)	294 (85.5%)
Yes	27 (14.8%)	23 (14.3%)	50 (14.5%)
**No MH history**			
No	71 (38.8%)	83 (51.6%)	154 (44.8%)
Yes	112 (61.2%)	78 (48.4%)	190 (55.2%)
**Medication History**			
**Antidepressants**			
No	124 (67.8%)	113 (70.2%)	237 (68.9%)
Yes	59 (32.2%)	48 (29.8%)	107 (31.1%)
**Antipsychotic**			
No	180 (98.4%)	154 (95.7%)	334 (97.1%)
Yes	3 (1.6%)	7 (4.3%)	10 (2.9%)
**Benzodiazepines**			
No	179 (97.8%)	153 (95.0%)	332 (96.5%)
Yes	4 (2.2%)	8 (5.0%)	12 (3.5%)
**Mood stabilizers**			
No	176 (96.2%)	154 (95.7%)	330 (95.9%)
Yes	7 (3.8%)	7 (4.3%)	14 (4.1%)
**Sleeping tablets**			
No	171 (93.4%)	150 (93.2%)	321 (93.3%)
Yes	12 (6.6%)	11 (6.8%)	23 (6.7%)
**Stimulants**			
No	177 (96.7%)	153 (95.0%)	330 (95.9%)
Yes	6 (3.3%)	8 (5.0%)	14 (4.1%)
**No medications**			
No	71 (38.8%)	59 (36.6%)	130 (37.8%)
Yes	130 (37.8%)	102 (63.4%)	214 (62.2%)
**Have you received MH counselling?**			
No	77 (42.1%)	90 (56.3%)	167 (48.7%)
Yes	106 (57.9%)	70 (43.8%)	176 (51.3%)
**Would you like to receive MH counselling?**			
No	18 (20.5%)	39 (41.9%)	57 (31.5%)
Yes	42 (47.7%)	22 (23.7%)	64 (35.4%)
Unsure/undecided	28 (31.8%)	32 (34.4%)	60 (33.1%)
**Clinical characteristics (scale used)**			
**ACE**			
**0**	28 (23.5%)	26 (26.3%)	54 (24.8%)
**1**	22 (18.5%)	12 (12.1%)	34 (15.6%)
**2**	17 (14.3%)	17 (17.2%)	34 (15.6%)
**3**	13 (10.9%)	11 (11.1%)	24 (11.0%)
**4 or more**	39 (32.8%)	33 (33.3%)	72 (33.0%)
**MDD (PHQ-9)**			
Unlikely MDD	34 (30.4%)	58 (59.8%)	9 (244.0%)
Likely MDD	78 (69.6%)	39 (40.2%)	117 (56.0%)
**GAD (GAD-7)**			
Unlikely GAD	50 (45.0%)	61 (62.9%)	111 (53.4%)
Likely GAD	61 (55.0%)	36 (37.1%)	97 (46.6%)

MH—mental health; PD—personality disorder; BD—borderline disorder; PTSD—post traumatic stress disorder; OCD—obsessive–compulsive disorder; ADHD—attention deficit hyperactivity disorder; SUD—substance use disorder; GAD—generalized anxiety disorder; MDD—major depressive disorder; PHQ-9—Patient Health Questionnaire-9; ACE-adverse childhood experience.

**Table 2:** Chi-square test of association between demographic, clinical, and mental health characteristics and likely generalized anxiety disorder.

Variables	Low Anxiety (GAD Unlikely) n (%) N=	High Anxiety (GAD Likely) n (%) N=	Chi-Square	*p* Value
**Sociodemographic characteristics**				
**Age (Years)**			6.62	0.01
≤26	50 (45.0%)	61 (55.0%)		
>26	61 (62.9%)	36 (37.1%)		
**Gender**				
Male	16 (48.5%)	17 (51.5%)		
Female	89 (54.3%)	75 (45.7%)	0.38	0.87
Other	6 (54.5%)	5 (45.5%)		
**Ethnicity**				
White	82 (52.6%)	74 (47.4%)		
Aboriginal	8 (50.0%)	8 (50.0%)		
Asian	10 (47.6%)	11 (52.4%)	* 4.10	0.40
African Descendants	2 (50.0%)	2 (50.0%)		
Other	9 (81.8%)	2 (18.2%)		
**Educational level**				
Less than high school	11(44.0%)	14 (56.0%)		
High school	17 (37.0%)	29 (63.0%)	8.73	0.01
Postsecondary education	83 (60.6%)	54 (39.4)		
**Relationship status**				
In a relationship (married, common law, partnered)	63 (66.3%)	32 (33.7%)		
Single	45 (43.3%)	59 (56.7%)	* 12.11	0.002
Separated/divorced/widowed	3 (33.3%)	6 (66.7%)		
**Employment status**				
Employed	67 (63.8%)	38 (36.2%)		
Unemployed	11(44.0%)	14 (56.0%)		
Student	21 (45.7%)	25 (54.3%)	9.82	0.02
Student and employed	12 (37.5%)	20 (62.5%)		
**Housing status**				
Own home	45 (72.6%)	17 (27.4%)		0.001
Rented accommodation	25 (44.6%)	31(55.4%)	13.12	
Live with family or friend	41 (45.6%)	49 (54.4%)		
**ACE score**				
**0**	35 (66.0%)	18 (34.0%)		
**1**	19 (61.3%)	12 (38.7%)		0.04
**2**	18 (56.3%)	14 (43.8%)	10.27	
**3**	10 (47.6%)	11 (52.4%)		
**4 or more**	26 (38.8%)	41 (61.2%)		
**MH history**				
**Depression**			12.14	0.001
No	80 (63.0%)	47 (37.0%)		
Yes	31 (38.3%)	50 (61.7%)		
**BD**			0.84	* 0.48
No	108 (54.0%)	92 (46.0%)		
Yes	3 (37.5%)	5 (62.5%)		
**GAD**			8.58	0.01
No	76 (61.8%)	47 (38.2%)		
Yes	35 (41.2%)	50 (58.8%)		
**Eating disorder**			0.09	0.81
No	102 (53.7%)	88 (46.3%)		
Yes	9 (50.0%)	9 (50.0%)		
**OCD**			4.85	0.03
No	105 (55.9%)	83 (44.1%)		
Yes	6 (30.0%)	14 (70.0%)		
**SUD**			0.37	* 0.67
No	109 (53.7%)	94 (46.3%)		
Yes	2 (40.0%)	3 (60.0%)		
**Schizophrenia**			1.77	* 0.50
No	109 (52.9%)	97 (47.1%)		
Yes	2 (97)	0 (0.0%)		
**PD**			1.79	* 0.26
No	109 (54.2%)	92 (45.8%)		
Yes	2 (28.6%)	5 (71.4%)		
**ADHD**			6.89	0.01
No	109 (55.6%)	87 (44.4%)		
Yes	2 (16.7%)	10 (83.3%)		
**PTSD**			0.36	0.56
No	96 (54.2%)	81 (45.8%)		
Yes	15 (48.4%)	16 (51.6%)		
**No MH history**			12.14	0.001
No	61 (67.0%)	30 (33.0%)		
Yes	50 (42.7%)	67 (57.3%)		
**Medication Hx**				
**Antidepressants**			10.32	0.002
No	88 (60.7%)	57 (39.3%)		
Yes	23 (36.5%)	40 (63.5%)		
**Antipsychotic**			0.04	* 0.99
No	107 (53.5%)	93 (46.5%)		
Yes	4 (50.0%)	4 (50.0%)		
**Benzodiazepines**			0.84	* 0.48
No	108 (54.0%)	92 (46.0%)		
Yes	3 (37.5%)	5 (62.5%)		
**Mood stabilizers**			2.69	* 0.15
No	109 (54.5%)	91 (45.5%)		
Yes	2 (25.0%)	6 (75.0%)		
**Sleeping tablets**			8.04	0.01
No	109 (55.9%)	86 (44.1%)		
Yes	2 (15.4%)	11 (84.6%)		
**Stimulants**			0.30	* 0.74
No	107 (53.8%)	92 (46.2%)		
Yes	4 (44.4%)	5 (55.6%)		
**No medications**			7.59	0.01
No	30 (40.5%)	44 (59.5%)		
Yes	81 (60.4%)	53 (39.6%)		
**Have you received MH counselling?**			2.01	0.17
No	59 (58.4%)	42 (41.6%)		
Yes	52 (48.6%)	55 (51.4%)		
**Would you like to receive MH counselling?**				
No	28 (93.3%)	2 (6.7%)		
Yes	15 (37.5%)	25 (62.5%)	22.69	0.000
Unsure/undecided	23 (53.5%)	20 (46.5%)		

* Fisher’s exact test.

**Table 3:** Logistic regression model for GAD.

	B	S.E	Wald	df	Sig.	Exp(B)	95% CI for EXP(B)	
							Lower	Upper
**Age (years)** ≤26	−1.348	0.859	2.461	1	0.117	0.260	0.048	1.400
**Education**								
Less than high school			0.910	2	0.635			17.943
High school	0.844	1.042	0.655	1	0.418	2.325	0.301	9.015
Post secondary education	0.662	0.784	0.714	1	0.398	1.939	0.417	
**Relationship status**								
In a relationship (married, common law, partnered)			1.652	2	0.438			
Single	−20.728	22974.557	0.000	1	0.999	0.000	0.000	
Separated/divorced/widowed	−19.963	22974.557	0.000	1	0.999	0.000	0.000	
**Employment status**								
Employed			3.330	3	0.344			
Unemployed	−1.225	0.969	1.598	1	0.206	0.294	0.044	1.963
Student	−0.098	1.040	0.009	1	0.925	0.906	0.118	6.962
Student and employed	0.100	1.061	0.009	1	0.925	1.105	0.138	8.840
**Depression disorder diagnosis? (Yes)**	−0.737	0.724	1.035	1	0.309	0.479	0.116	1.980
**Anxiety disorder mental health diagnosis (Yes)**	−0.387	0.843	0.211	1	0.646	0.679	0.130	3.547
**Obsessive–compulsive disorder diagnosis? (Yes)**	0.184	0.974	0.036	1	0.851	1.202	0.178	8.113
**ADHD diagnosis? (Yes)**	−0.278	1.607	0.030	1	0.863	0.757	0.032	17.663
**Medication**								
**On antidepressants, e.g., Prozac? (Yes)**	0.575	0.923	0.389	1	0.533	1.777	0.291	10.842
**On sleeping tablets, e.g., Zopiclone? (Yes)**	−1.240	1.455	0.726	1	0.394	0.289	0.017	5.009
**Would you like to receive mental health counselling?**								
No			12.109	2	0.002			
Yes	−3.290	1.104	8.880	1	0.003	0.037	0.004	0.324
Unsure/undecided	0.519	0.567	0.837	1	0.360	1.680	0.553	5.108
**ACE Score**								
0			3.673	4	0.452			
1	−0.910	0.710	1.643	1	0.200	0.403	0.100	1.618
2	−0.708	0.936	0.572	1	0.450	0.493	0.079	3.088
3	0.633	0.919	0.475	1	0.491	1.884	0.311	11.402
4 or more	−0.594	0.841	0.498	1	0.480	0.552	0.106	2.871
Constant	23.363	22,974.557	0.000	1	0.999	14,008,491,102.178		

**Table 4:** Chi-square test of association between demographic, clinical, and mental health characteristics and likely major depressive disorder.

Variables	At Most Mild Depression (MDD Unlikely) n (%) N=	Moderate-to-Severe Depression (MDD Likely) n (%) N=	Chi-Square	*p* Value
**Sociodemographic characteristics**				
**Age (Years)**			18.28	0.000
≤26	34 (30.4%)	78 (69.6%)		
>26	58 (59.8%)	39 (40.2%)		
**Gender**				
Male	14 (42.4%)	19 (57.6%)		
Female	74 (44.8%)	91 (55.2%)	0.35	* 0.90
Other	4 (36.4%)	7 (63.6%)		
**Ethnicity**				
White	69 (44.2%)	87 (55.8%)		
Aboriginal	7 (41.2%)	10 (58.8%)		
Asian	9 (42.9%)	12 (57.1%)	1.17	* 0.90
African Descendants	1 (25.0%)	3 (75.0%)		
Other	6 (54.5%)	5 (45.5%)		
**Educational level**				
Less than high school	6 (24.0%)	19 (76.0%)		
High school	12 (26.1%)	34 (73.9%)	15.23	0.000
Postsecondary education	74 (53.6%)	64 (46.4%)		
**Relationship status**				
In a relationship (married, common law, partnered)	56 (58.9%)	39 (41.1%)	16.51	* 0.000
Single	32 (30.5%)	73 (69.5%)		
Separated/divorced/widowed	4 (44.4%)	5 (55.6%)		
**Employment status**				
Employed	57 (54.3%)	48 (45.7%)		
Unemployed	12 (48.0%)	13 (52.0%)		
Student	15 (32.6%)	31 (67.4%)	12.32	0.01
Student and employed	8 (24.2%)	25 (75.8%)		
**Housing status**				
Own home	43 (69.4%)	19 (30.6%)		
Rented accommodation	19 (33.3%)	38 (66.7%)	22.96	0.000
Live with family or friend	30 (33.3%)	60 (66.7%)		
**ACE score**				
**0**	32 (60.4%)	21 (39.6%)		
**1**	15 (48.4%)	16 (51.6%)		
**2**	15 (46.9%)	17 (53.1%)	11.53	0.02
**3**	6 (28.6%)	15 (71.4%)		
**4 or more**	22 (32.8%)	45 (67.2%)		
**MH history**				
**Depression**			16.18	0.000
No	70 (55.1%)	57 (44.9%)		
Yes	22 (26.8%)	60 (73.2%)		
**BD**			1.22	* 0.47
No	90 (44.8%)	111 (55.2%)		
Yes	2 (25.0%)	6 (75.0%)		
**GAD**			17.70	0.000
No	69 (56.1%)	54 (43.9%)		
Yes	(26.7%)	63 (73.3%)		
**Eating disorder**			1.31	0.33
No	86 (45.3%)	104 (54.7%)		
Yes	6 (31.6%)	13 (68.4%)		
**OCD**			0.73	0.48
No	85 (45.0%)	104 (55.0%)		
Yes	7 (35.0%)	13 (65.0%)		
**SUD**			4.86	* 0.04
No	92 (45.3%)	111 (54.7%)		
Yes	0 (0.0%)	6 (100.0%)		
**Schizophrenia**			2.57	*0.19
No	90 (43.5%)	117 (56.5%		
Yes	2 (100.0%)	0 (0.0%))		
**PD**			0.70	*0.47
No	90 (44.6%)	112 (55.4%)		
Yes	2 (28.6%)	5 (71.4%)		
**ADHD**			3.87	0.05
No	90 (45.7%)	107 (54.3%)		
Yes	2 (16.7%)	10 (83.3%)		
**PTSD**			0.65	0.45
No	80 (45.2%)	97 (54.8%)		
Yes	12 (37.5%)	20 (62.5%)		
**No MH history**			15.35	0.000
No	54 (59.3%)	37 (40.7%)		
Yes	38 (32.2%)	80 (67.8%)		
**Medication Hx**				
**Antidepressants**			11.41	0.001
No	75 (51.7%)	70 (48.3%)		
Yes	17 (26.6%)	47 (73.4%)		
**Antipsychotic**			0.44	* 0.73
No	89 (44.5%)	111 (55.5%)		
Yes	3 (33.3%)	6 (66.7%)		
**Benzodiazepines**			0.14	* 0.99
No	89 (44.3%)	112 (55.7%)		
Yes	3 (37.5%)	5 (62.5%)		
**Mood stabilizers**			1.22	* 0.47
No	90 (44.8%)	111 (55.2%)		
Yes	2 (25.0%)	6 (75.0%)		
**Sleeping tablets**			7.42	0.01
No	91 (46.4%)	105 (53.6%)		
Yes	1 (7.7%)	12 (92.3%)		
**Stimulants**			0.001	* 0.99
No	88 (44.0%)	112 (56.0%)		
Yes	4 (44.4%)	5 (55.6%)		
**No medications**			10.24	0.001
No	22 (29.3%)	53 (70.7%)		
Yes	70 (52.2%)	64 (47.8%)		
**Have you received MH counselling?**			3.92	0.05
No	52 (51.0%)	50 (49.0%)		
Yes	40 (37.4%)	67 (62.6%)		
**Would you like to receive MH counselling?**			22.17	0.000
No	13 (31.7%)	28 (68.3%)		
Yes	13 (31.7%)	28 (68.3%)		
Unsure/undecided	19 (44.2%)	24 (55.8%)		

* Fisher’s exact test.

**Table 5:** Logistic regression model for depression.

Variables	B	S.E.	Wald	df	Sig.	Exp(B)	95% CI for	EXP(B)
**Age (years)** **≤26**	−0.379	0.840	0.203	1	0.652	0.685	0.132	3.554
**Education**	−							
Less than high school			3.492	2	0.174			
High school	2.184	1.345	2.639	1	0.104	0.113	0.008	1.570
Post secondary education	−2.302	1.254	3.370	1	0.066	0.100	0.009	1.168
**Relationship status**								
In a relationship (married, common law, partnered)			1.987	2	0.370			
Single	0.962	0.683	1.987	1	0.159	2.618	0.687	9.978
Separated/divorced/widowed	20.885	30,920.913	0.000	1	0.999	1,175,587,424.827	0.000	
**Employment status**								
Employed			0.523	3	0.914			
Unemployed	0.305	0.926	0.108	1	0.742	1.357	0.221	8.325
Student	0.518	1.127	0.211	1	0.646	1.678	0.184	15.281
Student and employed	0.718	1.131	0.403	1	0.525	2.051	0.223	18.823
**Depression disorder? (Yes)**	0.245	0.811	0.092	1	0.762	1.278	0.261	6.262
**Anxiety disorder diagnosis (Yes)**	1.134	0.928	1.493	1	0.222	3.108	0.504	19.160
**Substance use disorder diagnosis? (Yes)**	−0.420	38412.835	0.000	1	1.000	0.657	0.000	
**ADHD? (Yes)**	−0.649	2.167	0.090	1	0.764	0.522	0.007	36.511
**Antidepressants, e.g., Prozac? (Yes)**	0.739	1.151	0.412	1	0.521	2.094	0.219	19.983
**Sleeping tablets, e.g., Zopiclone? (Yes)**	21.785	15585.341	0.000	1	0.999	2,891,085,851.290	0.000	
**Have you received mental health counselling in the past? (Yes)**	−2.916	1.264	5.324	1	**0.021**	0.054	0.005	0.645
Would you like to receive mental health counselling?								
No			10.902	2	**0.004**			
Yes	3.690	1.123	10.797	1	**0.001**	40.026	4.432	361.505
Unsure/undecided	3.363	1.144	8.647	1	**0.003**	28.862	3.069	271.447
**ACE Scores**								
0			4.292	4	0.368			
1	−0.505	0.959	0.277	1	0.598	0.604	0.092	3.952
2	0.693	0.937	0.547	1	0.460	2.000	0.319	12.550
3	−0.116	0.996	0.013	1	0.908	0.891	0.127	6.271
4 or more	1.233	0.764	2.607	1	0.106	3.431	0.768	15.326
Constant	−2.184	1.653	1.745	1	0.186	0.113		

## Data Availability

Data for this study is available on reasonable request from the corresponding author.
